# Seroprevalence of *Burkholderia pseudomallei* among Adults in Coastal Areas in Southwestern India

**DOI:** 10.1371/journal.pntd.0004610

**Published:** 2016-04-14

**Authors:** Kalwaje Eshwara Vandana, Chiranjay Mukhopadhyay, Chaitanya Tellapragada, Asha Kamath, Meghan Tipre, Vinod Bhat, Nalini Sathiakumar

**Affiliations:** 1 Department of Microbiology, Kasturba Medical College, Manipal University, Manipal, Karnataka, India; 2 Department of Community Medicine, Kasturba Medical College, Manipal University, Manipal, Karnataka, India; 3 Department of Epidemiology, University of Alabama, Birmingham, Alabama, United States of America; Mahidol University, THAILAND

## Abstract

**Background:**

Although melioidosis, is an important disease in many Southeast Asian countries and Australia, there is limited data on its prevalence and disease burden in India. However, an increase in case reports of melioidosis in recent years indicates its endemicity in India.

**Aims and methods:**

A population-based cross-sectional seroprevalence study was undertaken to determine the seroprevalence of *B*. *pseudomallei* by indirect haemagglutination assay and to investigate the associated risk determinants. Subjects were 711 adults aged 18 to 65 years residing in Udupi district, located in south-western coast of India.

**Key results:**

Overall, 29% of the study subjects were seropositive (titer ≥20). Females were twice as likely to be seropositive compared to males. Rates of seroprevalence were similar in farmers and non-farmers. Besides gardening, other factors including socio-demographic, occupational and environmental factors did not show any relationship with seropositive status.

**Major conclusions:**

There is a serological evidence of exposure to *B*. *pseudomallei* among adults in India. While the bacterium inhabits soil, exposure to the agent is not limited to farmers. Non-occupational exposure might play an important role in eliciting antibody response to the bacterium and may also be an important factor in disease causation.

## Author Summary

Melioidosis is an underdiagnosed and underreported disease in India with protean clinical manifestation and high fatality. Causative agent *B*. *pseudomallei*, after environmental exposure, may cause disease or survive unnoticed for a long time in human and animal hosts. Individuals with diabetes and renal disease develop melioidosis with greater intensity and fatality. Recent increase in case reports of melioidosis in India might only be a tip of iceberg and represent small portion of a larger public health problem in the background of large number of diabetic population in the country. The paucity of data on disease prevalence has prompted us to undertake this sero-epidemiological study in a site located along the west coast of south India with heavy rainfall and rice farming as predominant agricultural activity. This site serves as a catchment for Kasturba Medical College and Hospital, Manipal, where nearly 20 to 30 cases of melioidosis are diagnosed each year. This first ever population-based seroprevalence study in India demonstrated a 29% seropositivity that is comparable with other endemic regions in Southeast Asia and Australia. Non-farmers were as likely to be seropositive as farmers. We also found a uniform seropositive status across various age groups and skill level of jobs. Females demonstrated higher seropositivity. Activities exposing the individual to environment such as gardening emerged as risk determinants of seropositivity.

## Introduction

*Burkholderia pseudomallei*, the etiological agent of melioidosis, is known to inhabit the soil and water in endemic areas in countries such as northeast Thailand, Singapore, Malaysia and the top end of Northern territory of Australia [1]. In these areas, the rural population involved in agricultural activities especially rice farming is at high risk of exposure primarily through inoculation, inhalation or aspiration. The host, once exposed, may harbor the bacteria for a prolonged period without any symptoms or may develop severe disease with protean manifestations, however the most likely outcome of exposure is seroconversion without harboring the bacterium at all [1–3]. While majority of patients present with community acquired pneumonia associated with sepsis, it is not uncommon for skin or soft tissue infections, multiple organ abscesses, neurological infections, bone and joint infection or pericardial effusion to occur [1, 2, 4]. Individuals with diabetes, renal disease or immunosuppressive illnesses suffer from more severe illness and relatively high mortality [1,2,5]. Several case reports or case series of melioidosis have been reported from India, including a large descriptive study that reported a high mortality of 41% in patients with septic shock [6–10]. It is possible that melioidosis is grossly under-diagnosed and/or misdiagnosed in India due to several factors such as lack of awareness amongst clinicians and microbiologists, absence of diagnostic laboratories in many rural areas, inadequate serological methods, inadequate surveillance systems, and limited research. In recent years, the increasing numbers of melioidosis cases reported across India, including our tertiary hospital located in the western coast of South India, strengthens the evidence of its endemicity in India [9,10]. Most of these patients are residents from the surrounding rural areas and present with late complications. Therefore, we undertook the present study to assess the rate of seroprevalence of *B*. *pseudomallei* and to identify the risk determinants of seropositivity among the adult general population residing in the catchment area of our hospital at Udupi.

## Methods

### Study design and population

A population-based cross-sectional study was conducted over a period of twelve months from June 2010 to May 2011. The study area was located in the coastal region of Udupi district along the west coast of Karnataka State, in south India, covering 929 square kilometer area at 13.3389° N and 74.7451° E. About 15% of the total population is engaged in agricultural activities primarily in rice farming, coconut and areca plantations. This region receives an average rainfall of 385 centimeters in a year during June to September from the southwest monsoon which is the main source of water for drinking and agricultural activities. Of the total 120 geographical divisions in Udupi, we randomly selected 23 (19%) locations and study subjects were sampled using probabilities proportional to their population sizes. After obtaining written informed consent, 711 adults between 18 and 65 years of age were recruited in the study. A field-tested questionnaire was administered face-to-face to elicit information on socio-demographics, occupation, activities leading to environmental exposure to the bacteria such as gardening, swimming etc., housing conditions, personal habits, lifestyle, travel and medical conditions. Serum samples were collected and stored at -70°C until tested. The serum samples were then assayed for the presence of anti-*B*. *pseudomallei* antibodies using indirect heamagglutination (IHA) test using the polysaccharide antigens prepared in our laboratory from clinical isolates of *B*. *pseudomallei*, as described by Peacock and Wuthiekanun [11]. Optimum dilution of antigen was titrated against the known positive serum provided by Mahidol Oxford Research Unit, Bangkok, Thailand. A titer of ≥20 was considered positive.

### Ethical approval

Ethical approvals were obtained from both the Institutional Ethics Committee of Manipal University, Manipal, India, and from the Institutional Review Board of University of Alabama at Birmingham, USA. Written informed consent for all procedures was obtained from the 711 adult subjects.

### Data analysis

All data including the heamagglutination titers were entered in Microsoft excel and analyzed using SPSS ver.16.0. Initial analyses compared subject characteristics according to seropositivity status using the Chi square test for categorical variables and t-test for continuous variables. Seropositivity was considered as a dichotomous variable (positive, tire level ≥20; negative, level <20). We analyzed association of various risk factors with prevalence of seropositivity using a Cochrane-Mantel-Haenszel statistics to estimate crude prevalence odds ratio (PORs) and their corresponding 95% confidence intervals (CIs). An exact logistic regression procedure was used for multivariable analysis to estimate adjusted PORs and their corresponding 95% CIs. A stepwise selection process was used to include significant variables in the multivariable models. We included a risk factor in a particular multiple regression models if the unadjusted POR was statistically significant or if the crude POR for the risk factor and seropositivity was ≥2 or ≤0.5 and if the prevalence of the seropositivity was at least 0.1 among subjects exposed to the risk factor. Statistical significance was considered with a p value less than 0.05. We also mapped the sampling sites and their corresponding rate of seroprevalence using ArcGIS 10.3. A cluster analysis was conducted to identify statistically significant hot spots, cold spots, and spatial outliers using the Anselin Local Moran's I statistic.

## Results

The overall seroprevalence (antibody tires ≥20) in the present study was 29% (n = 206). About 53% of subjects did not show any evidence of seropositivity while 18% had antibody titers of 10 thus, raising overall rate of any demonstrable antibody levels to 47%. Table 1 displays subject characteristics according to seropositivity status and the associated crude POR and CI for each potential risk factor. The median age of study participants was 36 years (SD ± 12.2, IQR 28–47 years). Seroprevalence did not vary significantly among the different age groups. Subjects below 50 years showed an average seropositivity of 29% and those above 50 years of age had a seropositivity of 26%. Women demonstrated higher seroprevalence compared to men (POR: 2.6, 95% CI: 1.86–3.62) while mean geometric value (MGV) of antibodies did not differ between the genders (31 in women and 32.8 in men). Furthermore, gardening and washing clothes in the river showed significant association with seropositivity but the latter had zero subjects in the seronegative group. There was no remarkable difference in seropositivity with regards to other risk factors such as occupation, job skill levels, activities involving environmental exposure, history of diabetes and other chronic disease. In final multivariate logistic regression analysis, only female gender emerged as an independent risk factor for seropositivity when adjusted for age, gardening and religion (Table 2).

**Table 1:** Number and proportion of subjects with potential risk factors by seropositive status.

Potential risk factor	Seropositivity^#^ (≥20) N	Seronegativity (<20) N	Crude POR** (95% CI)	P-value
**Total (N = 711)**	**206**	**505**		
**Gender**				
Male	84	324	1.0	<0.001
Female	122	181	**2.60 (1.86–3.62)**	
**Age**				
Mean* (SD)	37.79 ±12.38	37.52 ±12.16	-	0.785
Median	36.5	36.0	-	
**Age categories**				
≤30	70	170	1.0	0.895
31–40	57	139	0.99 (0.65–1.50)	
41–50	47	106	1.07 (0.69–1.67)	
>50	32	90	0.87 (0.53–1.42)	
**Major occupational groups**				
Famers	34	90	1.0	0.675
Non-Farmers	172	415	0.92 (0.59–1.42)	
**Skills level of jobs**				
Professional ^a^	15	31	1.0	0.543
Sales worker	8	25	0.66 (0.24–1.81)	
Production worker ^b^	48	144	0.68 (0.34–1.38)	
Agricultural worker	35	87	0.83 (0.40–172)	
Other worker	100	216	0.95 (0.49–1.85)	
Missing	-	2	-	
**Smoking**				
No	190	458	1.0	0.320
Yes	12	42	0.68 (0.35–1.33)	
Refused to answer	4	5	-	
**Alcohol consumption**				
No	182	425	1.0	0.350
Yes	15	52	0.67 (0.37–1.22)	
Refused to answer	9	28	-	
**Walking in fields**				
No	167	397	1.0	0.464
Yes	39	108	0.8 (0.57–1.29)	
**Work in the rice fields**				
No	179	419	1.0	0.194
Yes	27	86	0.73 (0.46–1.17)	
**Gardening**				
No	98	303	1.0	**0.002**
Yes	108	202	**1.71 (1.23–2.39)**	
**Wash clothes in the river**				
No	202	505	1.0	**0.002**
Yes	4	0	**4.09 ×10**^**9**^ **(0.0-∞)**	
**Wear footwear**				
Always	173 (29.2)	420	1.0	0.651
Sometimes	33 (28.0)	85	0.78 (0.12–3.86)	
**Swimming**				
No	169	400	1.0	0.392
Yes	37	105	0.84 (0.55–1.28)	
**Diabetes**				
No	187	433	1.0	0.026
Yes	4	24	0.38 (0.13–1.12)	
Do not know	15	48		
**Other comorbid conditions**				
No	186	442	1.0	0.449
Yes	6	17	0.83 (0.32–2.16)	
Do not know	14	46		

*Comparison using student’s t-test

**Crude Prevalence Odds ratio.

^#^ Seropositive status based on antibody titer levels. Titer levels ≥ 20 are considered seropositive.

^a^ includes technical administrative, and managerial occupations

^b^Includes skilled and unskilled manual occupations

**Table 2:** Prevalence odd ratio (POR) and 95% confidence intervals for seropositivity and potential risk factors, multiple logistic regression models.

Risk factor	Gender	Wash clothes in river	Gardening	Religion	Age^#^
**Model 1**	2.51				
POR* (95% CI)	1.70–3.53				
**Model 2**	2.61	3.3×10^9^			
POR* (95% CI)	1.07–6.36	0-∞			
**Model 3**	2.28	3.3×10^9^	1.21		
POR* (95% CI)	1.57–3.30	0-∞	0.83–1.75		
**Model 4**	2.01	3.02×10^9^	1.29	0.53	
POR* (95% CI)	1.37–2.95	0-∞	0.88–1.88	0.33–0.85	
**Model 5**	2.17	2.91×10^9^	1.41	0.50	Agecat 1: 1
POR* (95%CI)	1.46–3.23	0-∞	0.95–2.08	0.31–0.81	Agecat 2: 0.68 (0.44–1.07)
					Agecat 3: 0.69 (0.42–1.12)
					Agecat 4: 0.51 (0.29–0.87)

^#^Age categories: Category 1: ≤30 years, category 2: 31–40 years, category 3: 41–50 years, category 4: >50 years.

*POR for a risk factor adjusted for all other variables in the model.

**Fig 1:**
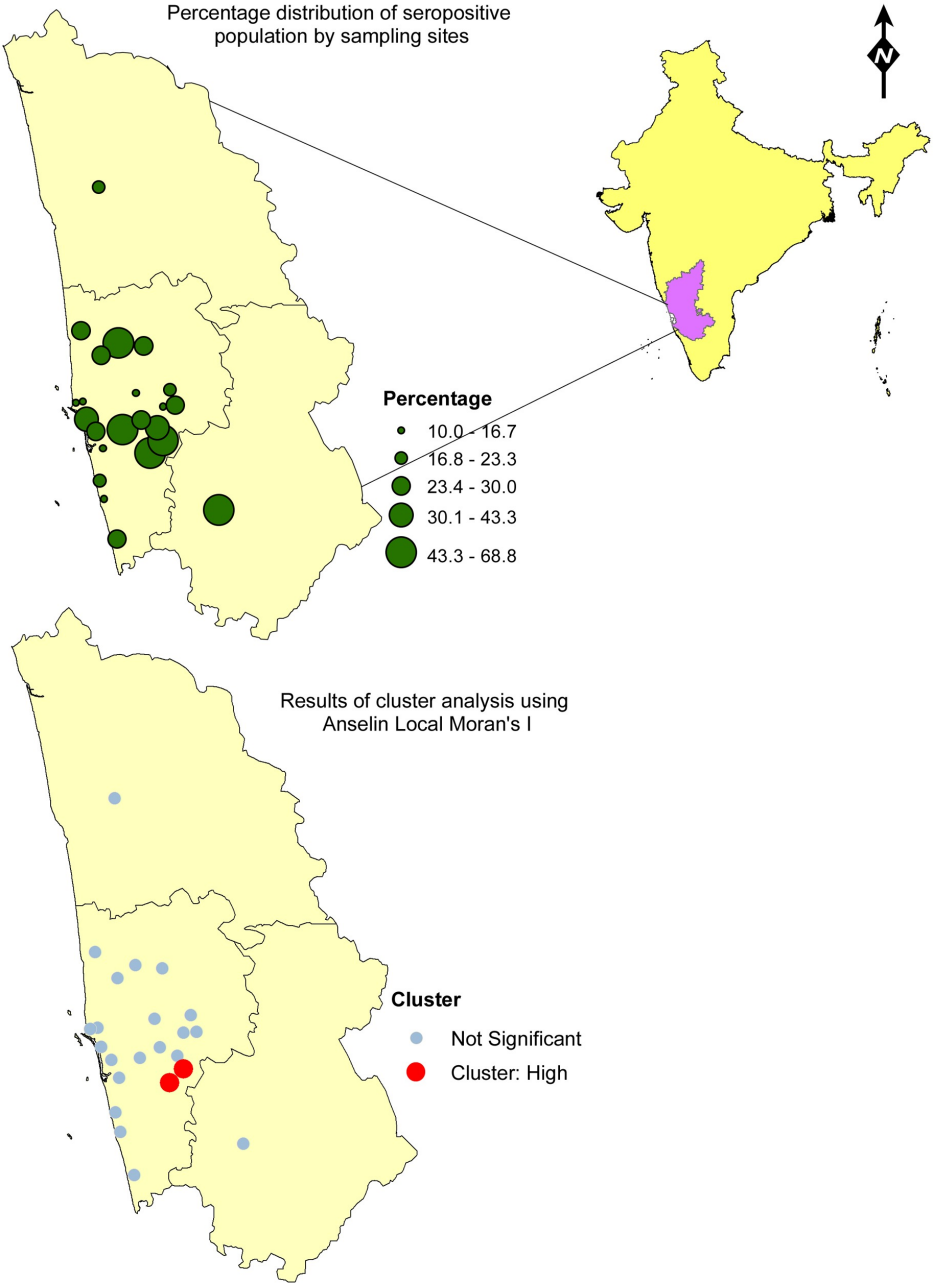
Mapping of proportion of seropositivity from sampled population in Udupi District in Karnataka India

Among various sampling locations, the rate of seroprevalence ranged between 10% - 69%. The results of the cluster analysis produced a map that displayed high rate geographical clusters (red) of seropositivity in the study area (Fig 1). The two sampling sites identified as clusters had seropositivity of 53.8% and 57.1%. We did not find any low rate clusters in the area. Based on our knowledge of the local geography, the clusters were found in close proximity to river beds where the land was used mainly for agricultural activities.

## Discussion

To our knowledge this is the first population-based survey from India to assess the serological evidence of exposure to *B*. *pseudomallei*. The present study showed 29% seropositivity to *B*. *pseudomallei* among the adult general population including farmers. Although there is no preferred method to study the exposure status of an individual to *B*. *pseudomallei*, IHA is the widely accepted method for this purpose [12]. An IHA titer of 1: 10 has been traditionally used as an evidence of exposure, but we analyzed results using a higher cut off ≥1:20 to enhance specificity of test results [13].

In bivariate analysis, risk factors including female gender, gardening and washing clothes in river, were found to be significantly associated with seropositive status. However, only female gender was found significant in multivariate analysis. Age and occupation, particularly those involving contact with soil and water did not show any relationship with seropositivity; in particular, differences in seropositivity status between farmers and non-farmers were unremarkable.

The overall rate of seroprevalence of 29% in the present study is comparable with rates reported from other endemic countries like Thailand (21–47%) [14], northern Australia (8–29%) [15] and Vietnam (6.4–31.8%) [16]. A previous study from a rice growing area near Vellore, South India, reported a seropositivity of 10.2% [17]. This evidence is further supported by detection of about 20 to 25 culture-confirmed cases of melioidosis every year at our single tertiary care center [9, 10].

The study results are important in light of conflicting results from countries with high incidence rates of melioidosis such as northeast Thailand and Northern Australia. Northeast Thailand shows high seropositivity and high incidence while the latter demonstrates lower seropositivity despite of high incidence rates of melioidosis [14, 18, 19]. Possible explanations for this difference include exposure to nonpathogenic cross reacting environmental *Burkholderia* species in Thailand leading to high seropositivity, differing clinical epidemiology between the regions, and speculations about differing modes of infection and virulence of bacterial strains. While the current understanding of clinical epidemiology in India suggest similarities in clinical presentations with both Northern Australia and Thailand [20], more research is warranted to explore the exposure dynamics of populations under study. India has the world’s largest population of >65 million people diagnosed with diabetes; about 25% of this population reside in rural areas. Diabetes is an established risk factor for melioidosis; thus, a large population is potentially at risk for melioidosis in rural areas who may be underdiagnosed or misdiagnosed [1, 21].

Among various socio-demographic factors examined, we found a significantly high seroprevalence in females compared to males although the disease has a male preponderance as reported earlier from a nearby tertiary care center [10] and in our experience. These results are consistent with several studies that also showed higher disease rates among males compared to females [12, 22]. In contrast, a recent report from Bangladesh described a similar seropositivity in both genders [23]. A difference in immunity to melioidosis between genders is unclear. It is speculated that the bulk of activities might determine the seroconversion among the genders. Less vigorous outdoor activities such as gardening or minor agricultural tasks with subsequent exposure to low inoculum of bacteria among women may lead to a mere serological response with or without subclinical infection, while men may be exposed to a larger inoculum leading to disease development due to their more rigorous outdoor physical activities. This hypothesis may be further supported by increased incidence of melioidosis during monsoonal season when individuals have prolonged and intense contact with soil and surface water at a higher inoculum of bacteria.

The relationship between age and seropositivity to *B*. *pseudomallei* has been inconsistent. Endemic countries such as Thailand reported an inverse relationship with age while a study from Bangladesh on non-melioidosis patients did not find any association with age [24,25]. Our study which excluded children < 18 years and did not find significant differences among adults in different age groups. The natural waning of antibody levels and reduced degree of exposure to *B*. *pseudomallei* might occur with increasing age although this could not be demonstrated in this study. The majority of the seropositive elderly in this study were actively working in rice fields which might have contributed for their positive antibody levels. It is currently unclear as to how long the antibody levels persist after environmental exposure to the bacterium. However, it is likely that continuous exposure to soil bacterium due to physical activities in adulthood may lead to demonstrable antibody levels in this age group.

We did not find any difference in seropositivity between farmers and non-farmers or among various skill levels of jobs. Activities involving environmental exposure to soil bacterium such as gardening, and washing clothes in river were positively associated with seropositive status across all occupational groups. This suggests that non-farmers are equally likely to be exposed to *B*. *pseudomallei* as farmers through activities that are not related to farm work. This finding gains prominence from a public health perspective in India as merely targeting specific occupational groups for disease surveillance or health education may not cover all population at risk of exposure to *B*. *pseudomallei*.

The spatial mapping showed high clusters of seropositivity in two of the 23 sampling locations. These sites were located about 10–15 kilometers from the coast and in close proximity to river bed with agricultural activity. Additional data from environmental sampling of soil and water in these areas could provide insights in to the relationship between level of bacterium in the environment and incidence of melioidosis cases.

The study had some limitations. It did not include children and hence, we could not determine the earliest age for seropositivity. Further, we could not correlate the clinical incidence rates of melioidosis and degree of seropositivity in various study locations as melioidosis disease mapping is still in its infancy in India. The IHA test detects antibodies against crude whole cell antigens of *B*. *pseudomallei*. Hence it is likely that some of the seropositivities may actually reflect cross reacting antibodies developed after exposure to *B*.*thailandensis*, a natural habitat of soil closely related to the former [26]. Due to limited resources, we could not expand the study to conduct environmental sampling of soil and water for presence of *B*. *pseudomallei* and other *Burkholderia* spp. that could have provided better insights into risk determinants of seropositivity.

The study adds valuable perspectives to the current understanding of melioidosis in this region in India. Awareness among primary physicians about the disease and strengthening laboratory facilities for early diagnosis would be the priority areas for capacity building. These findings prompt us to explore the ecology of *B*. *pseudomallei* in the environment before we conclude significant risk determinants of seropositivity. A detailed search for environmental distribution including soil and surface water combined with weather determinants would provide more insights into the burden of *B*. *pseudomallei* in the environment.

### Conclusion

This first seroprevalence study has provided important evidence of environmental exposure to *B*. *pseudomallei* in India where cases of melioidosis are increasingly detected. Although a low health priority at the current time, findings of this study reiterates the need to dedicate more resources to diagnosis, treatment and environmental research pertaining to meliodosis in India.

## Supporting Information

S1 Checklist STROBE checklist. (DOC)
